# Advances in *Entamoeba histolytica* Biology Through Transcriptomic Analysis

**DOI:** 10.3389/fmicb.2019.01921

**Published:** 2019-08-20

**Authors:** Sarah Naiyer, Alok Bhattacharya, Sudha Bhattacharya

**Affiliations:** ^1^School of Environmental Sciences, Jawaharlal Nehru University, New Delhi, India; ^2^School of Life Sciences, Jawaharlal Nehru University, New Delhi, India

**Keywords:** *Entamoeba histolytica*, transcriptome, virulence-related, encystation, promoter motifs, highly transcribed genes, gene expression regulation, downstream motifs

## Abstract

A large number of transcriptome-level studies in *Entamoeba histolytica*, the protozoan parasite that causes amoebiasis, have investigated gene expression patterns to help understand the pathology and biology of the organism. They have compared virulent and avirulent strains in lab culture and after tissue invasion, cells grown under different stress conditions, response to anti-amoebic drug treatments, and gene expression changes during the process of encystation. These studies have revealed interesting molecules/pathways that will help increase our mechanistic understanding of differentially expressed genes during growth perturbations and tissue invasion. Some of the important insights obtained from transcriptome studies include the observations that regulation of carbohydrate metabolism may be an important determinant for tissue invasion, while the novel up-regulated genes during encystation include phospholipase D, and meiotic genes, suggesting the possibility of meiosis during the process. Classification of genes according to expression levels showed that amongst the highly transcribed genes in cultured *E. histolytica* trophozoites were some virulence factors, raising the question of the role of these factors in normal parasite growth. Promoter motifs associated with differential gene expression and regulation were identified. Some of these motifs associated with high gene expression were located downstream of start codon, and were required for efficient transcription. The listing of *E. histolytica* genes according to transcript expression levels will help us determine the scale of post-transcriptional regulation, and the possible roles of predicted promoter motifs. The small RNA transcriptome is a valuable resource for detailed structural and functional analysis of these molecules and their regulatory roles. These studies provide new drug targets and enhance our understanding of gene regulation in *E. histolytica*.

## Introduction

*Entamoeba histolytica* is a protozoan parasite of humans, and causative agent of amoebiasis. The parasite exists in two forms; an infective, non-dividing cyst stage and an actively dividing, invasive trophozoite. The dormant, non-motile cyst, upon ingestion by the host, gets converted to the actively dividing trophozoite in the colon. Trophozoites rapidly divide by binary fission, get converted into cysts which are excreted in the feces and can cause fresh infection. Most infections remain asymptomatic, with only 5–10% being symptomatic (Watanabe and Petri, 2015) where the trophozoites invade the intestinal mucosa, resulting in rapid tissue damage and ulceration. The infection may spread to other organs, notably the liver, causing liver abscesses which may be fatal if left untreated. Estimations of the worldwide burden of amoebiasis indicate that approximately 500 million people are infected by the parasite and 10% of these individuals had invasive amoebiasis (Walsh, 1986; Shirley et al., 2018). The degree of pathogenesis of clinical isolates varies greatly. The factors that govern the outcome of infection, and adaptation of the parasite to host conditions are not clearly understood.

Gene expression studies have played a major role in understanding *E. histolytica* biology. More than 30 years ago the first cDNA library of this organism was prepared, and actin cDNA was sequenced (Edman et al., 1987; Huber et al., 1987) followed by other functionally important genes like ferredoxin, galactose-inhibitable lectin, superoxide dismutase and alpha tubulin (Huber et al., 1988; Tannich et al., 1991, 1992; Sanchez et al., 1994). In the pre-genomics era the first EST analysis helped to identify novel genes in *E. histolytica* (Azam et al., 1996).

Though studying and quantifying individual transcripts by northern blot and real time PCR has yielded useful gene level information in all organisms, the emergence of transcriptomics revealed complex landscape and dynamics of the transcriptome at an unprecedented level of sensitivity and accuracy (Ueda et al., 2004). The release of *E. histolytica* genome sequence and resources in 2005 (Loftus et al., 2005) spurred a series of genome-wide microarray gene expression profiling studies (Gilchrist et al., 2006; Weber et al., 2006; Baumel-Alterzon et al., 2013; De Cádiz et al., 2013; Thibeaux et al., 2013), and RNA-Seq analyses (Ehrenkaufer et al., 2013; Meyer et al., 2016; Naiyer et al., 2019) under a variety of conditions to understand the biology of the organism. The aim was to find answers to the important questions in amoebiasis: which parasite and/or host factors determine the outcome of *E. histolytica* infection ranging from asymptomatic cyst passer to invasive disease involving intestine, liver and other organs? How could trophozoite to cyst conversion be blocked to control disease transmission? How does *E. histolytica* cope with growth stress? Transcriptomic studies were used to monitor changes in gene expression in response to a variety of conditions, including tissue invasion, various stresses, drug treatments, encystation, and in virulent versus avirulent strains of *E. histolytica*. In addition, transcriptomic studies provided important information about the regulation of transcription itself.

**FIGURE 1:**
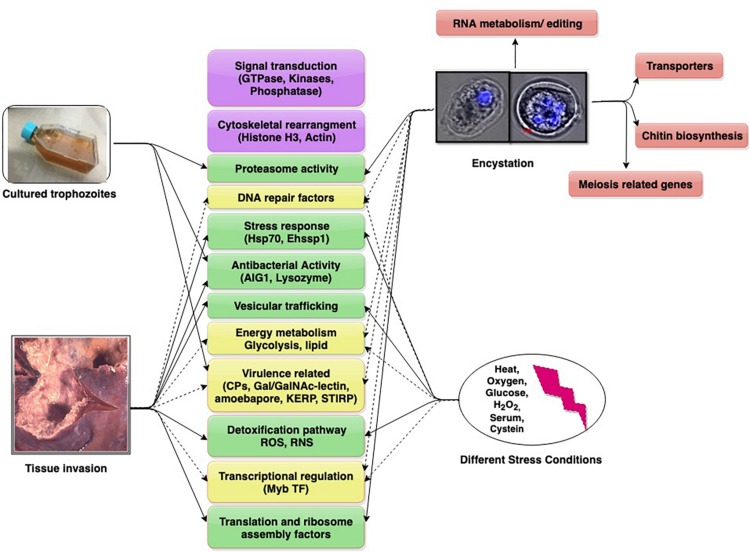
Differentially expressed genes/pathways revealed from transcriptomic date. The major pathways are shown in boxes (with gene in parenthesis). Pathways scored in all studies (purple), three studies (yellow, dotted arrows), two studies (green), single study (pink).

Individual *Entamoeba* labs have employed diverse models in these studies. Animal models used are gerbil liver, hamster liver, mouse colon, and human colon explants. Studies with cultured trophozoites have compared virulent (HM-1:IMSS) and avirulent (Rahman) strains of *E. histolytica*, as also pathogenic and non-pathogenic clones of HM-1:IMSS. This diversity has added to the complexity of data. We present and discuss contemporary findings in the field (obtained through transcriptomics) to understand the dynamics of important and common up/down regulated genes which may have roles in growth, survival, virulence, and infection. These genes/pathways may serve as potential future targets for amoebiasis control (Figure 1).

The genome of the protist parasite *E. histolytica* (Loftus et al., 2005; Lorenzi et al., 2010) provided clue to the development of new diagnostics and therapeutics for this disease. Since, *E. histolytica* does not form cysts in lab condition, a reptilian parasite *Entamoeba invadens* serves as a good model to investigate the differentiation process and drug targets.

## Using Transcriptomics to Study Differences Between Virulent and Avirulent *E. histolytica* Strains

### Cultured Trophozoites

The comparison of the transcriptome by whole genome microarrays of virulent and non-virulent *E. histolytica* has been a productive avenue of investigation for the identification of novel and detailed analysis of known, virulence determinants in *E. histolytica* like cysteine proteases, Gal/GalNac inhibitable lectin, amoebapores, lysozymes etc. Most *E. histolytica* infections are asymptomatic, with a small proportion (5–10%) resulting in invasive disease. The question has been posed whether *E. histolytica* strains of varying virulence potential exist, and if so, what are their important genetic differences? Of the *E. histolytica* strains studied in the lab, HM-1:IMSS is considered the prototype virulent strain, while the Rahman strain, isolated from an asymptomatic patient is much less virulent (Mattern et al., 1978). Transcriptomic analysis using a custom 70mer oligonucleotide-based microarray showed extensive differences between these two strains (Davis et al., 2007). The highly transcribed virulence-factor genes expressed in HM-1:IMSS included some of the cysteine proteases (CPs), Gal/GalNAc-inhibitable lectin light chain subunit (Lgl) involved in signal transduction, and the bacterial-killing molecule lysozyme, a protein with cecropin (antibacterial peptide) domain, and members of the AIG1 gene family, originally described in *Arabidopsis thaliana* as encoding GTPase domain-proteins involved in plant resistance to bacteria (Reuber and Ausubel, 1996). It was concluded that multiple pathways, involving signal transduction, antibacterial activity, cytoskeletal rearrangements, and protease production or secretion, possibly account for differences in virulence properties between the two strains.

HM-1:IMSS and Rahman strains belong to different genetic backgrounds. To overcome this limitation a study was undertaken with two cell lines, both derived from the virulent HM-1:IMSS strain. Cell line HM-1:IMSS-A had completely lost its ability to induce liver abscess in gerbils and mice, whereas cell line HM-1:IMSS-B induced large abscesses. Comparison of their transcriptomes revealed that only 19 genes showed a fivefold or higher differential expression in either cell line (Biller et al., 2009). Three rab7 GTPases were expressed more abundantly in the non-pathogenic cell line, while AIG1-like GTPases showed higher levels of transcription in the pathogenic cell line.

Further, to avoid complications due to cell lines containing a mixture of cells, twelve clones were obtained from cell lines A and B, and tested for ability to induce liver abscess (Meyer et al., 2016). Transcriptomic profiles of one non-pathogenic A-clone (A1np), one pathogenic B-clone (B2p) and one non-pathogenic B-clone (B8np) were determined. Only a few genes were differentially regulated when the two non-pathogenic clones A1np and B8np were compared with the pathogenic clone B2p, showing that different mechanisms may lead to loss of pathogenicity. An overexpression of a total of eight proteins in clone B2p were identified, including some hypothetical proteins, a metallopeptidase, C2 domain proteins, alcohol dehydrogenases, which may be important for regulating pathogenicity of *E. histolytica* (Meyer et al., 2016).

### Trophozoites Passaged in Animal Tissues

*Entamoeba histolytica* trophozoites grown in lab culture tend to lose virulence compared with those passaged in animals. RNA expression was compared between trophozoites from gerbil liver abscess and those in culture, using differential display PCR (Bruchhaus et al., 2002). Seven amplicons were up-regulated while five were down-regulated in abscess-derived trophozoites. The up-regulated genes included histone H3, ribosomal proteins RPS30 and RPL37A, cyclophilin, ferredoxin 2, and monomeric GTP-binding protein RAB7D. The diversity of these genes indicates that liver abscess formation would require the concerted action of a variety of proteins associated with stress response, signal transduction, regulation of translation and vesicular trafficking. Two genes specifically down-regulated in abscess-derived amoeba were flavoprotein and grainin1. Flavoprotein is known to detoxify intracellular RNS and/or ROS while grainin1 is found in intracellular granules and is postulated to be involved in the control of endocytic pathways and Ca^2+^ dependent granular discharge.

The first genome-wide analysis of *E. histolytica* transcriptome was carried out with trophozoites isolated from the colons of six infected mice on day 1 and 29 after infection versus *in vitro* cultured cells (Gilchrist et al., 2006). The up-regulated transcripts in colonic trophozoites were cell signaling molecules like trans membrane kinases, Ras and Rho family GTPases and calcium binding proteins. Significant decreases in mRNA abundance for glycolysis genes and increases in lipases were consistent with changes in energy metabolism. Defense against intestinal bacteria was suggested by alterations in AIG1 family transcription. Decreases in oxygen detoxification pathways were observed as expected in the anaerobic colonic lumen. Amoebic virulence-related molecules CP4-like proteins and Lgl were up-regulated.

The transcriptome of virulent HM-1:IMSS and avirulent Rahman strains was studied upon contact with human colon explants, using cDNA microarrays (Thibeaux et al., 2013). Within 1 h of contact with human colon, virulent trophozoites began to penetrate the mucus layer. The transcriptome of invasive amoeba showed expression of several virulence factors (the Gal/GalNAc lectin, STIRP, KERP1, and CP-A5) (Faust and Guillen, 2012). Other upregulated genes included the Myb family transcription factor (with SHAQKYF domain) involved in regulating genes in diverse pathways; genes linked to stress responses and signaling pathways, and the GTPase AIG1 (Thibeaux et al., 2013).

Most remarkably, the virulent strain showed up-regulation of genes for carbohydrate metabolism. On down-regulating the glycoside hydrolase (ß-amylase), mucus depletion and tissue invasion by virulent HM-1:IMSS was abolished (Thibeaux et al., 2013). It was concluded that when glucose levels in the colonic lumen are low, virulent *E. histolytica* might proficiently utilize host mucus glycans as carbon source, thereby initiating intestinal amoebiasis (Thibeaux et al., 2013). Whether this proficiency of *E. histolytica* to utilize host mucus glycans as carbon source determines its invasive potential is still to be demonstrated.

Transcriptomic changes of a single virulent amoebic strain were studied in four contexts, namely hamster liver abscess, human colon explants, long-term cultured virulence-attenuated cells, and short-term cultured trophozoites (Weber et al., 2016). Major transcriptome changes seen in virulent parasites upon contact with human colon explants led to the conclusion that activity of glycosylases, together with cytoskeleton and DNA repair activities, were the most important mechanisms underlying amoebic intestinal invasion. In long-term cultured parasites, the increase of proteasome activity and down-regulation of translational machinery (tRNA synthetases) was likely to alter the gene expression program (Weber et al., 2016).

**FIGURE 2:**
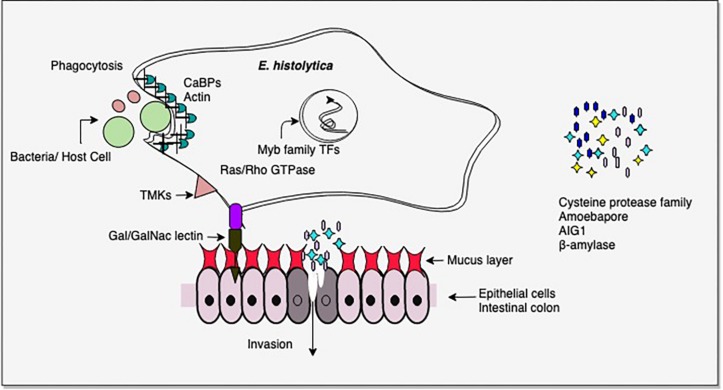
Important virulence-related molecules revealed from transcriptomic studies. Cysteine proteases and β-amylase assist in tissue invasion. β-amylase helps in mucus depletion and utilization. AIG1 functions in defense against intestinal bacteria and amoebapores mediate cell lysis. Contact of host cell ligands is facilitated by Gal/GalNAc-inhibitable lectin. Trans membrane kinases (TMKs) and Ras/Rho GTPases are involved in signal transduction. Myb family transcription factors regulate the expression of multiple pathways. Phagocytosis of bacteria/host cells requires the activity of a large number of proteins, including calcium binding proteins (CaBPs) which mediate actin reorganization. Please see text for details.

From the above studies on virulent versus avirulent strains, or invasive trophozoites versus lab grown cells, it is clear that the pathogenic phenotype in *E. histolytica* involves multiple pathways, and importantly, pathogenicity could be gained/lost through involvement of different sets of pathways under different conditions (Mattern et al., 1978; Reuber and Ausubel, 1996; Bruchhaus et al., 2002; Gilchrist et al., 2006; Davis et al., 2007; Biller et al., 2009; Faust and Guillen, 2012; Thibeaux et al., 2013; Meyer et al., 2016; Weber et al., 2016). Thus pathogenicity cannot be attributed to one or a few molecules. Nevertheless, some common factors do recur in many studies. The most common differentially regulated genes were in signal transduction pathways, carbohydrate metabolism, antibacterial activity (AIG1), virulence-related functions (CPs, Gal/GalNAc lectin), and transcription factors, notably Myb family. There were major changes in energy metabolism. In the intestine virulent *E. histolytica* may utilize host mucus glycans as carbohydrate source, thereby depleting the mucus and initiating invasive disease. AIG1 expression may be important in the colon as it is thought to be involved in antibacterial defense (Figure 2).

### Differential Gene Expression in *E. histolytica* Trophozoites Exposed to Virulence-Related Host Factors and During Phagocytosis

Attachment of *E. histolytica* trophozoites to collagen leads to subsequent tissue destruction and invasion (Serrano et al., 1996). To understand the underlying mechanism, gene expression changes were studied in collagen-activated trophozoites. A shotgun genomic DNA microarray containing 9600 random inserts from *E. histolytica* DNA was used (Debnath et al., 2004). The fourteen differentially up regulated clones obtained included signaling components, adapter proteins for vesicle formation and cytoskeletal reorganization and locomotion proteins. Virulence related cysteine proteases EhCP1 and EhCP2, and amoebapore were also up regulated. Thus this data, obtained from a relatively low-coverage microarray, highlighted the major virulence determinants required for tissue invasion. The same genomic microarray (Debnath et al., 2004) was further used to study other virulence-related factors. Intestinal invasion of *E. histolytica* trophozoites requires attachment to the colonic mucous layer, and cytolysis of host epithelial and inflammatory cells. The genomic array was probed with differentially labeled cDNAs, prepared from GalNAc-exposed, mucin-exposed, CHO-exposed and bacteria-exposed trophozoites (Debnath et al., 2007). The most abundant transcript up-regulated with GalNAc was the 170-kDa Gal/GalNAc lectin, followed by peroxiredoxin or thiol-specific antioxidant transcripts. The exposure of trophozoites to GalNAc-rich mucin may stimulate the interaction of lectin with thiol-specific antioxidants, which may protect *E. histolytica* trophozoites from oxidative attack during tissue invasion. The cysteine proteases, EhCP1 and EhCP2 were up-regulated threefold during *E. histolytica* interaction with mucin, which may be required for mucin cleavage. EhCP3 was down-regulated threefold during interaction with CHO cells. Genes up regulated during amoeba-bacteria interaction included protein kinase, ABC transporter, Rab family GTPase and hsp 90, showing adaptation to stress.

In another study (Sateriale et al., 2012), Affymetrix DNA microarray chips were used to analyze gene expression changes in response to phagocytosis. *E*. *histolytica* trophozoites are known to recognize human C1q protein, and phagocytose apoptotic Jurkat T lymphocytes opsonized with C1q (Jose et al., 2008). This property of C1q binding was used to sort phagocytic versus non-phagocytic trophozoite populations using C1q as a ligand. Differential gene expression in these populations was checked by microarray analysis (Sateriale et al., 2012). One hundred twenty one genes were found with >twofold higher expression in phagocytic than in non-phagocytic amoeba. These included genes known to be important for amoebic phagocytosis, e.g., those coding for proteins involved in actin binding and cytoskeletal organization, and genes with known roles in virulence, including two myosin heavy chain genes and a phosphatidylinositol phosphate kinase (Arhets et al., 1998). Exposure to host ligands correlates with colocalization of Gal/GalNAc lectin subunits in lipid rafts and phosphatidylinositol (Edman et al., 1987; Huber et al., 1987)-bisphosphate signaling in *E. histolytica* (Goldston et al., 2012). In addition, there was up regulation of helicase-like SNF2 domain proteins known to be involved in chromatin remodeling and structural maintenance of chromosomes. Interestingly, the authors speculate that up regulation of these genes could indicate preparation for cell division following phagocytosis. This study also showed enrichment of SH3 domain in upregulated genes of phagocytic *E*. *histolytica*. The important proteins in which this domain is commonly found include those involved in tyrosine kinase signaling, and proteins with putative bin/amphiphysin/Rvs (BAR) sequence. The latter are known to induce, stabilize, and sense membrane curvature (Frost et al., 2009) and could thus be important for phagocytosis. A technical limitation of this study was that it did not differentiate phagocytosis and cell adherence, and some of the effects could be due to adherence alone.

## Impact of Different Types of Stress Conditions on the *E. histolytica* Transcriptome

Tissue invasion by *E. histolytica* trophozoites entails an adaptive response to the host environment to ensure parasite survival. As a proxy to environmental adaptation in the host it would be instructive to study changes in *E. histolytica* in response to lab-generated growth stress. An oligonucleotide-based microarray analysis of *E. histolytica* exposed to heat shock (42°C, 4 h) revealed generally reduced transcription (Weber et al., 2006). Prominent down regulated genes were those for ribosomal proteins, and peroxiredoxin, the thiol-containing surface antigen. The small number of up-regulated genes (apart from HSPs), included chaperones, ubiquitination components involved in protein breakdown, the stress inducible gene Ehssp1 (Satish et al., 2003), and transcription factor TFIIIB required for transcription of small, untranslated RNAs.

*Entamoeba histolytica* trophozoites encounter cytotoxic reactive oxygen and nitrogen species in host tissues. A transcriptome study using whole-genome microarray was done with trophozoites exposed for 60 min to H_2_O_2_ or to a NO donor (DPTA-NON-Oate) (Vicente et al., 2009). Many genes were up- or down-regulated, but changes were not observed in the ROS and RNS detoxification pathways. This is perhaps because *E. histolytica* trophozoites constitutively express a number of scavenging enzymes even under basal conditions. Up-regulated genes belonged to diverse pathways including integrity of DNA, protein and lipids, signaling pathways involving protein kinases, phosphatases and acetyltransferases, and virulence-related genes. Interestingly, the non-pathogenic strain Rahman showed much decreased response to oxidative stress (Vicente et al., 2009). It could experience greater oxidative damage in the host, leading to reduced virulence.

Glutathione is the major thiol in eukaryotes. One of the metabolic features of *E. histolytica* is that it completely lacks glutathione and relies on L-cysteine as the major redox buffer (Nozaki et al., 2005). Transcriptomic changes were studied in L-cysteine deprived conditions, using a microarray (Husain et al., 2011). Functions associated with differentially expressed genes were metabolism, signaling, DNA/RNA regulation, electron transport, stress response, membrane transport, vesicular trafficking/secretion, and cytoskeleton. Interestingly, L-cysteine depletion did not significantly alter the expression of genes in sulfur-containing amino acid metabolism and in oxidative and nitrosative stress defense, which corroborates the study reported above (Vicente et al., 2009). Among the common genes up-regulated by L-cysteine deprivation and also by oxidative/nitrosative stress were those encoding iron sulfur flavoproteins, which have proposed functions in oxidative stress (Cruz and Ferry, 2006).

During invasive growth *E. histolytica* trophozoites can migrate to the liver, which is considered a much more glucose-rich environment than colonic lumen. Transcriptomic changes in *E. histolytica* adapting to low glucose were studied using a microarray (Baumel-Alterzon et al., 2013). The up-regulated genes included the surface antigen ARIEL, CP4, Gal/GalNAc lectin, lysozyme, amoebapore, Ras protein family transcript, amylase, dihydro pyrimidine dehydrogenase (DPD), and amino acid degrading enzymes. DPD, which catalyses pyrimidine degradation, may be important as it was required for parasite survival in the colon as well (Gilchrist et al., 2006).

Nutritional stress due to serum starvation is known to affect expression of individual genes (Shrimal et al., 2010, 2012; Ahamad et al., 2015). RNA-Seq analysis was done to look at global regulation of transcription in serum-starved cells (Naiyer et al., 2019). Maximally up-regulated genes were in signaling pathways (kinases and GTPases), lipid metabolism, DNA repair factors, translation factors, Myb family transcription factors, BspA family, HSPs and cell cycle regulators. Genes showing maximum down-regulation coded for some signaling factors (Rab/Ras/Rho), energy metabolism, actin and actin-binding proteins, Ariel family, and ribosome biogenesis factors. The down-regulation of metabolism related transcripts and actin-binding proteins is in keeping with reduced energy requirement and cell motility during serum starvation.

Taken together, the transcriptomic changes in response to different stresses showed that, as expected, growth-related genes and actin binding proteins were down regulated in keeping with reduced energy requirement and cell motility, while some of the chaperones, and ubiquitination components for protein breakdown were up regulated. A common consequence of several stresses is DNA damage; hence up regulation of DNA repair pathways was frequently observed. Up regulation of TFIIIB is interesting as it is needed for transcription of small untranslated RNAs which may be key players in modulating the gene expression changes to cope with stress. Apart from transcriptional changes, there was evidence that post-transcriptional or post-translational mechanisms might also be involved.

## Transcriptomic Changes in Trophozoite to CYST Conversion: *E. invadens* as a Model

*Entamoeba* cysts can withstand environmental pressure and are responsible for disease transmission. Drugs that inhibit encystation can, therefore, break the transmission cycle. Encystation of *E. histolytica* trophozoites has not been achieved in axenic lab culture, although the same is possible for the reptilian parasite *E. invadens*, which is used as a model system to study stage conversion.

The first transcriptomic analysis of *E. invadens* encystation, using DNA microarrays, was done in a time-course study after transfer to encystation medium. Genes associated with metabolic pathways were studied, and metabolomic profiles were monitored by mass spectrometry (Jeelani et al., 2012). As expected from cells preparing to enter dormancy, the levels of glycolytic pathway intermediates, and transcripts of corresponding genes were significantly depleted. Conversely, metabolites in chitin biosynthesis, and expression of their genes increased after 8 h. Glycogen/starch biosynthesis decreased, whereas their breakdown increased. The glucose thus released may be utilized for chitin synthesis (Jeelani et al., 2012).

Up-regulation of an aspartate aminotransferase suggested that aspartate may be used as energy source when glucose is not available. Levels of biogenic amines went up during the early stages of encystation, while polyamines like spermidine and spermine decreased (Jeelani et al., 2012). Interestingly, at later stages of encystation the levels of γ-aminobutyric acid (GABA) increased, which may be physiologically significant, as GABA has been reported to induce sporulation in the slime mold *Dictyostelium discoideum* (Anjard, 2006). This study showed a close correlation between changes in metabolic profiles and transcription of genes in the corresponding pathways.

Global transcriptional changes during encystation and excystation of *E. invadens* were determined by RNA-Seq analysis of 11,549 genes (Ehrenkaufer et al., 2013). There was general down-regulation of genes associated with translation and ribosome assembly, while nuclear proteins associated with nucleosome assembly were up-regulated, ostensibly to package DNA in a silent configuration in cysts. In general, the reverse trend was seen during excystation. In early encystation (8–24 h) many genes were up-regulated, while fewer genes were down-regulated, relative to trophozoites. This pattern was reversed in 48 and 72 h cysts where many genes were down-regulated. Genes up-regulated early in encystation included signaling molecules such as protein kinases, small GTPase-activating and lipid-signaling proteins, transcriptional regulation, and Myb (SHAQKY) transcription factors, genes in RNA metabolism, for example RNAse P, and the RNA-editing protein pseudouridine synthase (Ehrenkaufer et al., 2013). Whether RNA editing is, indeed involved in *Entamoeba* development and encystation would be an intriguing possibility.

In mature cysts (72 h), down-regulated genes included those encoding basic metabolic processes, as seen by Jeelani et al. (2012), and up-regulated genes included RNA binding proteins, which may be part of ribonucleoprotein structures (chromatoid bodies) found in *Entamoeba* cysts (Barker, 1964). DNA repair and chromatin assembly genes were up-regulated in mature cysts which are engaged in nuclear division (Singh et al., 2011). Phospholipase D involved in lipid second messenger signaling was up-regulated in cyst (Ehrenkaufer et al., 2013), and its activity was required for encystation but its mechanism of action is still to be studied. It was also up-regulated in *E. histolytica* cysts (Ehrenkaufer et al., 2007a). Another interesting finding from this study was that meiosis-related genes like MND1, Hop2, and Rad52 were up-regulated at 24 h. Although meiosis has not been demonstrated in *Entamoeba*, homologous recombination does take place, and homologs of meiotic genes exist (Singh et al., 2013). Studies with *Giardia intestinalis* strongly indicate that DNA exchange occurs following nuclear fusion during encystation (Poxleitner et al., 2008). The equivalent process in *Entamoeba* needs to be demonstrated.

In another transcriptomic study of *E. invadens* encystation using DNA microarrays (De Cádiz et al., 2013), the up-regulated genes encoded metal ion transporters, cytoskeletal proteins, vesicular trafficking (small GTPases), Myb (SHAQKY) transcription factors, some CPs, components of proteasome, and enzymes for chitin biosynthesis. Genes involved in cytoskeletal rearrangement were regulated at the early phase when trophozoites start aggregating. Another up-regulated gene was BspA, a leucine-rich repeat-containing, abundant multicopy gene implicated in attachment and invasion of host cells (Davis et al., 2007). Serine/threonine protein phosphatases were up-regulated at late stages. Most genes in energy metabolism were repressed (Davis et al., 2007). However, several genes involved in nucleotide, lipid and sphingolipid metabolism continued to be transcribed at late stages, in agreement with earlier studies (Jeelani et al., 2012; Ehrenkaufer et al., 2013). Table 1 enlists the important pathways involved in encystation.

**TABLE 1:** Important pathways in encystation.

**Stage**	**Pathway**	**Up/down**	**Remarks**
Early	Glycolysis	Down	Preparation for dormancy
Early	Translation, ribosome assembly	Down	Preparation for dormancy
Early	Signaling molecules	Up	Reprograming
Early	Cytoskeletal rearrangement	Up	Trophozoite adherence
Early	Chitin biosynthesis	Up	Cyst wall initiation
Early	Nucleosome assembly	Up	Silent chromatin
Early	Myb transcription factor	Up	Transcriptional regulation
Early	RNA metabolism	Up	Post transcriptional
Late	Carbohydrate, lipid metabolism	Down	Dormancy
Late	RNA-binding proteins	Up	Chromatoid bodies
Late	DNA repair	Up	Nuclear division
Late	Meiosis	Up	DNA exchange

Data from above studies taken together show that early in encystation the transcriptomic changes were those expected from cells preparing to enter a dormant state (Barker, 1964; Davis et al., 2007; Hackney et al., 2007; Vicente et al., 2009; Jeelani et al., 2012; Singh et al., 2013). The transcript levels of glycolytic pathway genes significantly dropped, and there was down-regulation of genes associated with translation and ribosome assembly. Conversely, genes involved in cytoskeletal rearrangement needed for trophozoite aggregation were up regulated. Other up regulated genes included those in chitin biosynthesis required for cyst cell wall, genes associated with nucleosome assembly required to package DNA in a silent configuration, and signaling molecules. In mature cysts, carbohydrate and lipid metabolism genes were down regulated, while some RNA binding proteins, which may be involved in formation of chromatoid bodies, were up regulated. Interestingly, meiosis-related genes like MND1 and Hop2 which bind to DNA at double strand breaks, and Rad52 which promotes recombination were up regulated at 24 h of encystation. Transcriptomic data has helped to identify key regulatory genes that are essential for encystation. Studies on individual genes are needed for discovery of new drug targets that inhibit stage conversion of the parasite.

## Effect of Amoebicidal Drugs, and Iron Metabolism on *E. histolytica* Transcriptome

Metronidazole (MTZ) has been the standard drug of choice for treatment of amoebiasis. Unlike *Trichomonas* and *Giardia*, *E. histolytica* cells resistant to high concentrations of the drug were difficult to culture and could be obtained in the lab only after continuous selection with increasing amount of drug in ∼200 days (Wassmann et al., 1999). Transcriptomic changes were monitored in cells resistant to 12 μM MTZ (Penuliar et al., 2015). The significantly up-regulated genes were DNA polymerase, iron-sulfur flavoprotein (ISF), and some members of AIG1 family. Another up-regulated gene was dUTPase, implicated in resistance to cancer chemotherapy. Up-regulation of DNA polymerases may impart plasticity needed for adaptation (Penuliar et al., 2015), and ISFs may enhance cell survival.

Auranofin, an FDA-approved drug for rheumatoid arthritis, was found to have potent amoebicidal activity (Debnath et al., 2015). Transcriptional profiling of cells treated with 1 μM auranofin suggested that the target of auranofin was likely to be thioredoxin reductase, inhibition of which would enhance the sensitivity of trophozoites to reactive oxygen-mediated killing (Arias et al., 2007).

Iron metabolism may be important for amoebic pathogenesis since iron levels are relatively low in the intestinal lumen compared to blood and liver (Espinosa et al., 2009). Trophozoites cultured in low-iron medium show reduced cytotoxicity (Lee et al., 2008). A transcriptomic comparison was done with trophozoites grown in normal versus low-iron medium (Hernández-Cuevas et al., 2014). The differentially expressed genes included transporters (homologous to bacterial siderophores and heme transporters) and genes involved in heme biosynthesis (GluRS and SAMS). In eukaryotes, iron can be recovered from heme via the action of heme oxygenase-1 (Kim et al., 2011). A monooxygenase was strongly down-regulated in iron-deficient trophozoites supplemented with hemoglobin, possibly to avoid the production of ROS and Fe^2+^ via heme catabolism (Tronel et al., 2013). *E. histolytica* may use this monooxygenase to recover iron from heme internalized after erythrophagocytosis.

These studies have provided important leads which will help to further explore the biochemical mechanisms involved. MTZ-resistance may involve up regulation of genes for ISFs. Some of the differentially expressed genes in iron-deficient conditions showed IRE-like structures. However, iron-regulatory protein homologs that work with IRE elements have yet to be identified in *E. histolytica*.

## Transcriptomic Analysis of Epigenetic Regulation in *E. histolytica*

Gene expression can be epigenetically regulated through cytosine DNA methylation, or through specific histone modifications. Transcriptomic analysis of cells treated with 5-Azacytidine (AzaC), an inhibitor of cytosine DNA methylation revealed two subsets of genes: (1) silenced by genomic DNA methylation and derepressed by AzaC, and (2) methylated but not silenced by methylation (Ali et al., 2007). The study concluded that DNA methylation had relatively limited effects on gene expression in *E. histolytica*, as transcription of only 2.1% genes was significantly modulated by AzaC. No association of gene silencing due to DNA methylation in the vicinity of retrotransposons EhLINEs/SINEs was found. Direct measurement of cytosine DNA methylation also showed lack of correlation between methylation and EhLINE1 silencing (Agrahari et al., 2017). In addition, the *E. histolytica* HSP70 promoter remained methylated during heat shock when the gene was actively transcribed (Agrahari et al., 2017). Thus, cytosine methylation may have a limited role in *E. histolytica* gene expression, as is also reported for *D. discoideum* (Kuhlmann et al., 2005).

The role of histone acetylation in *E. histolytica* gene expression was studied using the inhibitor Trichostatin A (TSA) (Ehrenkaufer et al., 2007b). No substantial overlap was observed between genes modulated by TSA and those by AzaC, indicating that histone modification and DNA methylation do not significantly crosstalk in *E. histolytica*. Interestingly, the genes regulated by TSA overlapped substantially with genes regulated during encystation, suggesting potentially important role of histone acetylation in regulating stage conversion.

The important conclusions from these inhibitor studies include the demonstration that DNA methylation had relatively limited effects on gene expression in *E. histolytica*, and that histone acetylation should be considered an important player in regulating stage conversion.

## Use of Transcriptome Data in Promoter Analysis and Transcription Regulation

Transcriptome data can be applied for analysis of gene regulatory features, and to understand transcript architecture. The first study to identify promoter motifs associated with high or low gene expression in *E. histolytica* used microarray data (Hackney et al., 2007). Conserved promoter motifs were identified by MEME, and a Bayesian classifier revealed groups of motifs significantly predictive of gene expression levels. A single motif (consensus sequence A/TAAACCCT), similar to an enhancer element in *Schizosaccharomyces pombe* (Matsumoto and Yanagida, 1985; Oliva et al., 2005), was predictive of high gene expression. It was enriched in the promoters of ribosomal proteins, and tRNA synthetases. Three motifs, associated with low gene expression, seemed to function synergistically as genes with ≥2 motifs had extremely low expression. Majority of these genes belonged to the *E. histolytica* stress sensitive protein (Ehssp) family (Satish et al., 2003) and were transcriptionally up-regulated during heat shock. This study used microarray expression data, which typically have a lower dynamic range of gene expression than RNA-Seq. In addition, only gene upstream sequences were analyzed for identification of promoter motifs.

**FIGURE 3:**
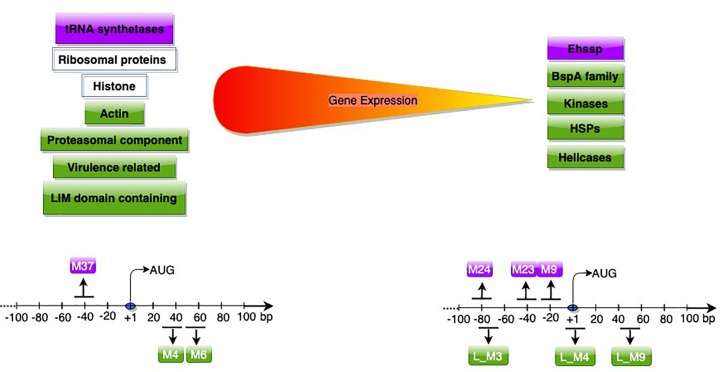
Genes (upper panel) and promoter motifs (M) (lower panel) associated with high/low gene expression. Genes and motifs in purple (Hackney et al., 2007) in green (Naiyer et al., 2019), in white (both studies). The peak position of each motif with respect to AUG is shown; for further details of dispersed motifs please refer to Naiyer et al. (2019).

A subsequent study used RNA-Seq data, and included sequences downstream of start codon to study promoter motifs associated with gene expression (Naiyer et al., 2019). Based on Log_2_ TPM (transcripts per million), genes were classified into expression classes. Interestingly, apart from genes expected to be highly expressed, (translation-related, oxidative stress, cytoskeletal functions); many of the virulence-related genes like amoebapore A, B, and C precursors, Gal/GalNAc lectin light subunit and the cysteine protease isoform, EhCP-A5 (Meyer et al., 2016), were also highly expressed in axenic culture. These genes are possibly required for optimal *E. histolytica* growth, and are additionally utilized for pathogenesis. Of seven motifs associated with high gene expression, two were associated only with highly expressed genes, and were required for gene expression. These novel motifs were located downstream of start codon (Figure 3). Downstream promoter elements, first reported in *Drosophila*, are commonly associated with TATA-less promoters (Burke et al., 1998; Juven-Gershon and Kadonaga, 2010). However, such correlation was not observed in *E. histolytica*, pointing to mechanistic differences. The mechanism of action of these downstream motifs and their association with high expression is yet to be explored.

In another study, the promoters of 57 amoebic genes that showed increased transcription in response to H_2_O_2_ exposure were analyzed (Pearson et al., 2013). A H_2_O_2_ regulatory motif (HRM), AAACCTCAATGAAGA was enriched within 100 nts of the start codon in these genes. An HRM DNA-binding protein was identified, and shown to increase basal expression. Overexpression of this protein resulted in increased virulence.

An upstream regulatory element, URE3 (TATTCTATT) has earlier been defined in *E. histolytica*, and its binding protein URE3-BP is a membrane-associated calcium responsive regulator of virulence genes Hgl5 and Fdx1 (Gilchrist et al., 1998). To identify additional genes regulated by URE3-BP, transcriptome profiling of a strain overexpressing URE3-BP was done. It revealed 50 transcripts, of which 8 were up-regulated while 42 were down-regulated (Gilchrist et al., 2008). Fifteen of the URE3-BP regulated genes were membrane proteins, highlighting the role of URE3-BP in *E. histolytica* surface remodeling and regulation of cellular motility in response to a calcium signal.

Alternative splicing and polyadenylation are known mechanisms which generate multiple isoforms from precursor mRNAs, thereby generating diverse phenotypes (Keren et al., 2010). While some of the alternative isoforms may be physiologically regulated, others may be derived from the inherent stochasticity of RNA processing. Hon et al. (2013) utilized RNA-Seq data to study the extent of alternative splicing and polyadenylation in *E. histolytica* and quantified the extent of stochastic noise. Most of the poly(A) sites fell within 100 nt downstream of stop codon, as also suggested in an earlier study (Zamorano et al., 2008). They identified a large number of rarely spliced alternative junctions and reported that alternative exon skipping, and intron creation are more likely to occur in abundant transcripts. However, they found the functional impact of these processes to be limited to a small proportion of genes, with most of the microheterogeneity likely to arise from stochastic events.

Small RNAs are known to be involved in antisense gene regulation in *E. histolytica* (Zhang et al., 2011). Pyrosequencing was done to identify the entire population of Argonaute 2-associated 27nt small RNAs (Zhang et al., 2013). These mapped to 216 supercontigs covering 52% of the *E. histolytica* genome. Further, 19 supercontigs contained ∼50% of all sequenced small RNAs. It is not known whether these 19 supercontigs belong to centromere or telomere regions. Overall, 358 protein coding genes had more than/equal to 50 small RNAs mapping to each other including 226 genes with only antisense RNAs, 45 genes with antisense and sense RNAs and 87 genes with only sense strand RNAs. Gene families with antisense RNAs included AIG1 family proteins, beta-amylase, deoxyuridine 5′-triphosphate nucleotide hydrolase domain proteins, DNA polymerase and C2 domain proteins. Most antisense small RNAs mapped toward the gene 5′-termini, which is similar to that in *Ascaris* (Wang et al., 2011). About 10% of differentially expressed genes in HM-1:IMSS versus Rahman strains could be due to small RNAs (Zhang et al., 2011). A notable example was EhSTIRP known to be highly expressed in HM-1:IMSS. 519 small RNAs mapped antisense to this gene in Rahman small RNA dataset compared to virtually none in HM-1:IMSS. This study demonstrated the depth of small RNA-mediated regulation in *E. histolytica*.

Thus, transcriptomic data provided new information about regulatory features of *E. histolytica* transcription, and the nature of highly expressed genes. This data has for the first-time revealed promoter motifs associated with high and low gene expression, including novel downstream motifs. These could be used to modulate the expression of genes introduced into *E. histolytica* by transfection and will aid further studies on basic understanding of promoter structure in *E. histolytica.*

## Conclusion and Future Prospects

Transcriptomics has provided valuable overall insights into the pathways and genes actively involved in adaptation of *E. histolytica* to a variety of conditions mimicking its natural life cycle. These data are a useful resource for further detailed analysis of individual and inter-connected pathways, and of specific genes that were not earlier suspected of novel roles. The significance of these data will be further enhanced as the putative functions of “hypothetical proteins” get revealed. Regulation of *E. histolytica* genes by small RNAs is another aspect that needs to be explored. While transcription of a gene is the primary and potent step at which gene expression is controlled, it is one of many steps that together determine the final availability of functional gene product. The extent to which post-transcriptional regulatory mechanisms operate in *E. histolytica* gene expression is not yet understood, although in many instances they seem to be important (Gupta et al., 2012; Ahamad et al., 2015). *E. histolytica* might employ a number of post-transcriptional mechanisms like RNA transport, protein modifications, allosteric regulations, and redirection of metabolic fluxes, to modulate phenotypic expression (Husain et al., 2011). Thus, transcriptomic data need to be correlated with proteomic and metabolomic data to better understand cell physiology and obtain a more holistic view of *E. histolytica* biology. Moreover the high transcription of virulence-related genes during normal parasite growth raises the question of their roles in processes other than virulence alone. The mechanism by which downstream promoter motifs associate with highly transcribed genes is still to be elucidated.

## Author Contributions

SN wrote the first draft of the manuscript. All authors contributed to the manuscript revision, read, and approved the submitted version.

## Conflict of Interest Statement

The authors declare that the research was conducted in the absence of any commercial or financial relationships that could be construed as a potential conflict of interest.
